# Linking risk communication and sustainable climate change action: A conceptual framework

**DOI:** 10.4102/jamba.v11i1.703

**Published:** 2019-07-15

**Authors:** Tom E. Volenzo, John O. Odiyo

**Affiliations:** 1 School of Environmental Sciences, University of Venda, Thohoyandou, South Africa

## Introduction

Combating climate change is one of the critical goals under the Sustainable Development Goals (SDGs) agenda. This is on account that several of the targets under the climate change action overlap and impinge targets envisioned under other SDGs in itself a social policy dilemma. The overlapping targets include the need to consider environmental impacts and sustainability outcomes on land and land-based resources, ecosystems and biodiversity as well as agricultural productivity and incomes of small-scale farmers. Transforming the socio-ecological systems towards sustainability is thus critical. While risk communication has successfully been applied in emergency management, especially the crisis phase, little of the concept has been adapted in natural resource management. Using the case of smallholder farmer adaptation to climate change, this article explores how risk communication can resolve cognitive failure among multiple actors in climate change action in the context of adaptation mitigation-sustainable development frameworks. Combined with communication for development approaches, risk-communication-mediated pathways have the potential to enhance risk assessment and development of multi-hazard early-warning systems in climate change action. In this regard, risk communication offers a seamless support system for the integration of the precautionary and disaster risk reduction principles in the pursuit of sustainable development agenda.

Climate change and weather variability are among the biggest challenges to human development as they present a combination of risks that negatively impact on human health, global food security, economic development and the natural environment on which much of the human livelihoods depends (Zakarya et al. 2015). Accordingly, combating climate change and its impacts are at the core of the 17 Sustainable Development Goals (SDGs) agenda (UN 2015). This is on account that several of the targets under climate change action overlap and impinge other SDGs. Risk reduction is thus advocated in addressing disaster risk drivers, such as poor land management, unsustainable use of natural resources and declining ecosystems, in climate change action and pursuit of SDGs (UNISDR 2015).

The pillars of sustainable development are anchored on economic, social and ecological dimensions (Kates, Parris & Leiserowitz 2016). Exploring socio-ecological linkages in sustainable development is particularly critical with the rising need for inclusion of climate-related targets in SDGs and a more climate-oriented set of indicators as part of systems for sustainable development and environmental quality (Opschoor & Banuri 2007). Such reality creates the need to reassess traditional policy instruments in terms of their adaptability to better reflect climate-related externalities of production and consumption. In this regard, a multi-hazard and multi-sectoral approach is envisaged in fostering people centred collaborative partnerships, mechanisms and institutions for implementation of instruments relevant to building resilient socio-ecological systems. This includes the sustainable management of natural resources (UNISDR 2015).

Though sustainable development thinking has benefitted immensely from a number of conceptual frameworks such as the nexus model (FAO 2014) and environment-livelihood sustainability (Biggs et al. 2015) models, existence of autonomous adaptation and maladaptation (Adger, Arnell & Tompkins 2005; Barnett & O’Neill 2010; FAO 2010a) and attendant ecological risks suggest failure by policy makers, farmers and community of practice to recognise the underlying drivers of risks in adaptation planning. Maladaptation occurs when adaptation action or investment taken to avoid or reduce climate change impacts increases vulnerability to other risks that impact adversely on, or increases the vulnerability of other systems, sectors or social groups (Barnett & O’Neill 2010). Maladaptation thus imposes negative externalities on third parties and ecosystems. Accordingly, meaningful integration against maladaptation is a challenging task in adaptation planning (Mimura et al. 2014). An externality is present when the production or consumption activities of one economic agent have direct, non-price-mediated effects on the production or consumption activities of another economic agent (Coase 1960).

The utility of any integrative model in a dynamic socio-ecological environment to a great extend depends on its visibility and coherence (Lang & Rayner 2012). Accordingly, in an attempt to bridge the gap between science and policy, increased attention on transdisciplinary paradigm and its variants have been advocated (Lang et al. 2012). However, most of the existing transdisciplinary models are short in addressing cognitive failure. Though existing models, such as the nexus model, identifies relationships and interdependencies in environmental resource management, it fails to explain how risk and behaviour, compromise and negotiation can be achieved in creating interdependencies at planning and policy phases (Biggs et al. 2015).

Mitigation of disasters requires active public participation and strong political mobilisation, which may be achieved through assessment of community perceptions, experiences and responses to disasters (Wisner, Blaikie & Davis 2004). Implicit in participation is multiplicity of actors with the tendency to generate coordination failures (DEA 2013). This calls for innovative social-political-ecological and legal processes, adjustment of institutional capacities and processes especially in relation to deliberations about risk appraisal (Stirling 2005). As the extent to which opinions and interests of stakeholders are taken into account are important in sustainability discourses (Del Rio & Burguillo 2009), we attempt to explore how cognitive dissonance of various actors, such as farmers, extension workers and policy makers in adaptation planning, can be addressed.

Climate Change communication can be an effective and sustainable means to mainstreaming of climate change into development policies, mitigation and adaptation polices, collective behavioural change and specifically positive attitudes towards climate change mitigation (Evans, Dyll & Teer-tomaselli 2018). Incorporating participatory communication and communication for structural and sustainable social change at different levels of society have been found to be critical in adaptation processes, climate change and environmental communication, as well as natural resource management (Servaes & Lie 2013). Communication media and communication for development approaches are thus recognised as critical platforms for engagement and empowerment in social and structural change such as those concerning adaptation and mitigation of climate change risks (Evans et al. 2018).

Dissemination of information or successful risk communication increases people’s knowledge and motivates the public and households to prepare and mitigate disasters (Dennis et al. 2011). Though risk communication has potential in the raising awareness about environmental and health risks, it has several limitations. Such limitations include risk of information overload (O’Neill 2002) and a high likelihood of targeted people failing to pay attention (Renn 2006). Further, the linearity in the coding, transmission and decoding can lead to substantial disengagement and the generation of meaning of the disseminated risk messages, distortion and redundancy (Jaeger & Renn 2001). The utility of risk communication in the construction of collective knowledge and attitudes towards climate change, a long-term sustained crisis, is thus debatable (Servaes & Lie 2013). Linearity or one-way communication is a form of passive participation that limits the extent to which a community or individuals can influence a programme (Arnstein 1969; Evans et al. 2018; Servaes & Lie 2013). One-way communication depresses learning and alienates intended recipients of communication (Fischhoff 2005).

In this article, we explore how the weakness in risk communication can be resolved and subsequently harnessed in the development of a ‘software’ for mitigation of maladaptation risks. In doing so, we draw on the concept of integrated structural and participatory model for climate change communication (Evans et al. 2018) to suggest a robust risk communication approach that can be adopted to promote sustainability in adaptation planning. Following the participatory model for climate change communication (SPCCC) model, we posit that integration of risk communication with communication for development approaches can reduce the risk of maladaptation and contribute to resilience building at community level. Though media, local culture, empowerment, agency and communication are critical in sustainable development (Servaes & Lie 2013; Evans et al. 2018), the scope of this article does not cover the diversity of communication media.

The central contribution of this conceptual article is a robust participatory communication framework that integrates risk communication and communication for development in the assessment of the nature, magnitude and significance of environmental risks, as well as the control, monitoring and evaluation of existing and underlying maladaptation risks in climate change action. By incorporating knowledge, attitudes and behaviour and addressing linearity weaknesses associated with risk communication, the model provides the basis for a holistic approach in the design and integration of multi-hazard early-warning systems into climate change adaptation planning.

### The review context

Adaptation to climate change is reflected in social, ecological and economic changes that mitigate the adverse impacts and take advantage of arising opportunities (Easterling et al. 2007). Adaptation to climate risks involves technical measures and modifications to farm practices with respect to climatic and non-climatic stimuli and conditions (Wall, Smit & Wandel 2004). Irrigation or the artificial application of water to supplement or compliment the available moisture for plant growth in response to prolonged dry spells, erratic rains or poorly distributed rains is one of the most common adaptation measures in agriculture. However, adaptation may or may not succeed in moderating harm or exploiting beneficial opportunities because system transformations are more challenging than planning or implementing immediate measures to cope with a climate-driven disaster (Moser & Ekstrom 2010).

Consideration for behavioural dynamics in the transformative processes is integral to social learning and sustainability agenda (Pelling 2011). Transformational agenda seeks to address the root cause of vulnerability by introducing fundamental changes to attributes of a system (Eriksen, Nightingale & Eakin 2015). Rethinking and reframing of policy and practice, engaging multiple knowledges as well as questioning subjectivities inherent in discourses and problem understanding are critical in pursuit of transformational agenda (Eriksen et al. 2015). Such analytical frameworks are inclusive of individual, community, state and non-state actors’ interests, aspirations and interactions. Building upon the foregoing argument, the rising need for resilience building to climate-related hazards (UN 2015), the importance of identifying underlying risk factors in risk reduction initiatives (UNISDR 2015) and the centrality of strategic planning, monitoring and evaluation advocated under comprehensive conceptual model for disaster management (Asghar, Alahakoon & Churilov 2005), we explore some concepts in disaster risk reduction in the next section.

Disaster risk management involves strategic planning (Asghar et al. 2005), administrative decisions and operational activities in the prevention, response, recovery, mitigation and early-warning for disasters (Satya 2010). Though the administrative activities are cross-cutting and a shared responsibility among various actors, the administrative decisions in agriculture are largely policy related while operational activities are largely farmer dependent. Risk reduction and resilience building at community level require analytical and conceptual lenses that unbundle cognitive biases and failures as well as integrate and transform individual and collective agency (Volenzo & Odiyo 2018). In farmers’ use of irrigation as an adaptative measure to climate change, the critical question is on how to transform communities and individuals to collectively internalise the salinisation risks.

The crunch-release model (Blaikie et al. 1994) is a broad analytical framework on the causes of a disaster and disaster mitigation. The model espouses factors influencing vulnerability to a disaster by considering trigger events, elements at risk of damage or loss and vulnerable conditions both socio-ecological and physical, against actions that address underlying causes to achieve safe conditions or resilience. Developmental advocacy, policy and legislative actions, risk assessments, multi-hazards early-warning systems, public awareness and education and training (Satya 2010) are among the disaster prevention and mitigation actions suggested in the model.

Disaster risk reduction is underscored as an overarching pillar for attainment of SDGs (UN 2015). Accordingly, there is need for improved understanding of disaster risks in all its dimensions of exposure, vulnerability, hazard characteristics, strengthening of disaster risk governance for prevention, mitigation, preparedness, recovery and rehabilitation. Though precautionary principle is a tool of choice for disaster risk reduction concerning health and environmental threats in an environment of scientific uncertainty, there is paucity of appropriate frameworks for integrating implementation and decision-making support processes in the application of the principle (Fisher, Jones & von Schomberg 2006).

Most of the natural resource management technologies, such as irrigation, have both larger spatial scales and longer time horizon impacts or externalities (Knox, Meinzen-dick & Hazell 1998). Failure to recognise and/or discount such threats constitutes cognitive failure and imposes long-term social costs on society (Coase 1960). The socio-ecological dilemma is rooted in isolation paradox (Baumol 2004) and free rider (Hardin 1968) analogies. This is particularly critical in planning adaptation to climate change because existence of strong environmental and conservation polices and legislation in many countries does not necessarily imply effective implementation and enforcement (Shivakoti, Ullah & Pradhan 2016). Designing cost-effective collective action strategies required for abatement of environmental externalities inherent in climate change action is thus a development planning challenge.

### Ethical considerations

This article followed all ethical standards for a research without direct contact with human or animal subjects.

## Analytical framework

Stakeholder attributes such as agency play essential role in the proactive and reactive approaches to disaster risk management (Mojtahedi & Oo 2017). This includes factors such as perception of risk, habit, social status and age which operate at individual and consequently collective action decision-making levels (Adger et al. 2009). Cognition or knowledge about risks is thus critical in risk reduction planning. Accordingly, approaches in climate change action need to integrate indigenous knowledge with latest scientific insights, address multiple stressors and trade-offs, build adaptive management capacity as well as focus on information management, sustainability, monitoring and evaluation of risks (DEA 2013; UN 2015; UNISDR 2015). However, individual adaptation hinges on whether an impact, anticipated or experienced, is perceived as a risk and whether it should (and could) be acted upon through adaptation policies, or is constrained by inertia and cultures of risk denial (Adger et al. 2009). As existing models are inadequate for effective public participation (FAO 2014), there is need for a hybrid climate change communication model that integrates bottom-up and top-down processes, embraces information and communication technologies (ICTs), empowers local communities and nurtures collective action (Evans et al. 2018).

Proactive engagement of stakeholders in early phases of disaster risk management is a requisite for resilient societies and built environment against disasters (Mojtahedi & Oo 2017). As adaptation is a continuous stream of activities, actions, decisions and attitudes that inform all aspects of life that reflect existing social norms (Nelson et al. 2007), it is important to frame it from social system lenses, the structure of which is provided by various individuals and groups of which it is composed (Rogers 2004). This is critical because social relations influence expectations, commitment and understanding of risks by individuals and communities (Kasperson et al. 1988). As the functional element within a social system is the potential or actual behaviour of the individual and the collectives in a social system in terms of knowledge, attitudes and behaviour, there has been a shift from diffusion and transfer of technologies to a broader understanding of how to involve multi-stakeholders through communication activities (FAO 2010b; Servaes & Lie 2013). This could be extended to holistic approaches that apply feedback loops between the climate system, the human system and ecosystems in assessment of adaptation planning (David & Elise 2007). Effective communication of the risks is thus posited to play an important role in smallholder farmers’ adaptation to climate change.

The characteristics that impact risk perception and behaviour and the degree of dread associated with the risk vary with the public’s familiarity with the risk (Slovic 2000). As hazards interact with psychological, social and cultural processes in ways that can heighten or attenuate individual and social perception of risk and shape risk behaviour (Renn et al. 1992), debates concerning societal values and world views require good understanding of the social context on which risk management decisions are made (OECD 2002). More critical is the observation that collective action influences individual action in climate change risk management (David & Elise 2007). As individual and social characteristics, in particular risk perception, interact with underlying values to form subjective and mutable limits to adaptation that currently hinder society’s ability to act, it could preclude adaptation at societal scales (Adger et al. 2009). This creates the need for integration of social learning and communication concepts in adaptation planning (Servaes & Lie 2013).

Under risk perception normalisation theory (Becker, Ronan & McClure 2017), people tend to think that they are not at risk from an existing risk. Perceptions of being safe may, however, change to perceptions of being at risk immediately after a disaster by providing a window of opportunity to motivate preparedness and mitigation. The impact of seeing what other people have performed to prepare and mitigate a disaster is a stronger motivation for taking action than receiving information about the need to take action (Mileti & Darlington 1997). Diffusion of technology models (Rogers 2004) could be used in partly explaining such observation. Diffusion innovation theory holds that observability is critical to adoption processes. In this way, people can bring direct, indirect and vicarious experiences to bear on their risk assessments and preparedness decision-making (Becker et al. 2017).

Risk-based decision-making results into balanced judgement that reflects factual evidence about the matter at hand in relation to interests and values, including messages on emerging risks that are currently not of public concern (Renn 2006). In principle, mobilising and influencing risk dispositions and behaviour of individuals through education and disclosures may transfer the agency of environmental and health risks control and enforcement from the state to individual (Rose 2003). In this way, risk communication offers a better alternative to regulatory enforcement. This may be utilised in the prediction, prevention and remediation of environmental and health risks (Wardman 2008). Implicitly, risk communication is a cost-effective alternative to regulatory systems in the abatement of environmental and health risks, and integral to effective decision-making in climate change action and sustainable development discourses.

While the inherent post-disaster emergency relief planning approach that is implicit in the risk normalisation bias theory (Becker et al. 2017) is relevant in reinforcing positive risk behaviour, it stands at crossroad with proactive approaches advocated by the disaster risk reduction paradigm. It is also at variance with precautionary principles in the management of environmental risks. According to the isolation paradox (Baumol 2004), individuals are willing to make a sacrifice on their present consumption (through abatement of ecological degradation risks) to benefit future generations if others do so. However, under the free rider analogy (Hardin 1968), individuals expect benefits from other people’s abatement initiatives, yet they are unwilling to do the same. As the agency of safeguarding and enforcing public interest through abatement falls on the government enforcement and compliance systems and institutions (Rose 2003), the relevance of the risk communication, particularly the governability model, is critical.

## Contextualising the critical issues

### Adaptation as disaster risk reduction pathway among farmers under changing climate

Climate change is the change in the state of climate whether because of natural variability or as a result of human activity that can be identified by changes in the mean and/or the variability of its properties, and that persists for extended period, typically decades or longer (IPCC 2007). Evidence of climate change includes changes in patterns of temperature and precipitation, irregular and un-predictable rainfall, intense downpours, rising temperature and generally increase in frequency of extreme and harsh weather such as droughts (IPCC 2014). In realisation of the negative impacts of climate change, urgent action to combat climate change through adaptation has been proposed (UN 2015). Accordingly, adaptation is a key factor that influences the future severity of climate change impacts (Easterling et al. 2007). Adaptation to climate change takes place through adjustments to reduce vulnerability or enhance resilience in response to observed or expected changes in climate and associated extreme weather events. It involves changes in social and environmental processes, perceptions of climate risk, practices and functions to reduce potential damages or to realise new opportunities. Adaptation includes anticipatory and reactive actions (Adger et al. 2007).

Maladaptation is underlying risk in climate change action (Adger et al. 2005; Barnett & O’Neill 2010; FAO 2010a). Hence, there is a need to investigate policies, plans and programmes in terms of risks and opportunities critical in sustainable development agenda. Underlying risks refer generally to the socio-economic and environmental factors that are the primary causes of a disaster. Individual and societal stocks of knowledge, attitudes, skills and the consequent risk behaviour are among the key factors that influence vulnerability (Satya 2010). Nuanced against the urgency for adaptation action in an environment of cognitive failure, the critical question is on who has the power to act or the ‘agency’ in safeguarding public good (interests). To answer the question requires an understanding of how hazards (trigger events), vulnerability and capacity interact. Models for such analysis are provided by Mimura et al. (2014) and Turner et al. (2003). In the current article, we contextualise the risks of salinisation from irrigation among small-scale farmers.

Salinisation is a major impediment in many arid and semi-arid regions of the world (Qadir, Qureshi & Cheraghi 2007). The global extent of primary salt-affected soils is about 955 million hectares (ha), while secondary salinisation affects some 77m ha, with 58% of these in irrigated areas. Salinisation is attributed to irrigation of unsuitable soils or use of poor-quality irrigation water.

For example, of the 34m ha of land under cultivation in Iran, 4.1 million ha is affected by salinisation. This represents $1 billion in annual economic losses (Qadir et al. 2007). The need for increase in area under irrigation in adaptation to and mitigation of adverse climate change impacts on food and forage production and thus presents a serious challenge to sustainability of irrigated production systems. This calls for risk reduction initiatives.

### Irrigation ecological sustainability linkages

Though definitions of sustainability vary across sectors, their common theme is to change the way resources are exploited, and how hazards are managed so that adverse impacts downstream or for subsequent generations are reduced (Kates et al. 2005). Sustainable development principles call for integration of economic and development policies so that in case of conflict between the two, ecological interests are given preference (UNEP 2012). The ratio of total factor productivity to changes in the critical soil property, such as salinity (magnitude of soluble salts in the soil) or the total natural resource productivity (TNRP), is used in the current article to contextualise the impact of irrigation on sustainability. As a coefficient or index of sustainability, TNRP accounts for indirect costs from degradation of a natural resource (Lal 1997).

Water quality and its suitability for use in irrigation are judged on potential severity of problems that can be expected to develop during its long-term use (FAO 1985; Ravikumar & Somashekar 2012). The most important determinants of quality and suitability of water for irrigation are total concentration of soluble salts (salinity hazard) in terms of electro-conductivity (EC), relative proportion of sodium to other principal cations (sodium hazard) expressed as sodium adsorption ratio (SAR), bicarbonate concentration relative to the concentration of calcium plus magnesium and boron hazards or concentration of boron or other toxic elements (Ravikumar & Somashekar 2012). Salinity hazards or EC exceeding certain threshold levels reduce water availability to crop in the root zone. Salinity hazards above such thresholds could cause 8%– 86% loss in crop yields (FAO 1985).

### The role of communication in the transformation of socio-ecological system

The most attractive adaptation measures are those that offer benefits in the near future as well as reduce vulnerabilities in the long-term (Mimura et al. 2014). Hence, adaptation to climate change requires supportive policy environment and support systems for resilience building. A supportive environment includes policies that improve access to quality services, leadership that promotes social and behavioural change among members of society, allocation of resources for social and behavioural change, community contribution to the implementation of solutions in general and support of individuals’ own behavioural change (FAO 2010b). This considers the interaction of individuals’ knowledge and behaviour on social change.

Participation, a central tenet in development planning discourses, refers to the inclusion of those who are affected or who can affect a decision (Hobley 1996). Rowe and Frewer (2000) conceptualise participation from a two-way flow of communication in terms of information dissemination to passive participants and gathering information from participants. The rationale for participation in natural resources management, however, is largely informed by local resistance to state control of natural resources and need to address tragedy of the commons arguments (Bixler et al. 2015).

Several authors (EEA 2014; Arnstein 1969) have reviewed broad spectrum of forms of public participation. According to some of the findings (Chambers 1994; Scoones 1998), participation improves the quality of decision-making process and improves use of available information and creativity in the society. Moreover, participation improves public understanding of the management issues at stake, enhances transparency in decision-making and might stimulate better coordination, monitoring and evaluation among government agencies. Participation increases better understanding, dialogue, out of thinking discipline, nuanced focus on relevant problems that confront the real drivers of change and formulation of strategy and policy (Mostert et al. 2007).

Empowerment refers to the capacity of people to make effective choices, envision alternatives, participate in decision-making, negotiate with influence, control and hold accountable institutions that affect their lives and livelihoods (World Bank 2007). Accordingly, empowerment is central to community-based disaster risk reduction and resilience planning initiatives (UNISDR 2015) with communication being at the core of empowerment, participation and nurturing collective responsibility in climate change decision-making processes (Evans et al. 2018; Servaes & Lie 2013). The underlying philosophy in communication for development is built around the principle of empowerment in the identification of problems, development of solutions and implementation strategies, monitoring and evaluation (FAO 2010b). This includes use of communication in extension education and research as a tool for improving productivity, incomes, welfare and sustainable natural resources management outcomes among farmers. For example, a democratic participatory approach that incorporates the interests, capacities and cultural identity of local communities at all levels has the potential to unpack the limitations of traditional media in empowering the community (Servaes & Lie 2013).

Communication for development is defined as planned use of strategies and processes of communication in achieving development and behaviour change (Srampickal 2006). The three basic components of communication for development are advocacy, social mobilisation and behavioural change (or behavioural development) communication. The other components include participatory communication and communication for structural and sustainable social change (Servaes & Lie 2013). Effective communication relies on the synergistic use of the three strategic components (UNICEF 2008). Advocacy informs and motivates leadership to create a supportive environment to achieve programme objectives and the related development goals. In essence, the advocacy component is aimed at changing policies, allocating resources, public dialogue and conversation on critical issues. Climate change action and sustainable development agenda are some of the cross-scalar issues.

Advocacy, sustained publicity and education can converge value systems to reduce gaps in knowledge, attitudes and beliefs (FAO 2010b) as well as translate existing adaptive capacities into adaptation actions that reduce vulnerability (Mimura et al. 2014). The backbone of advocacy whether national or at the local level comes from a combination of data analysis and community input (UNICEF 2008). Possible results of an advocacy intervention can be targeted at leaders taking actions, such as legal reform, or enactment of new law(s), or rules, policy decisions, formulation of and/or reform, administrative directives resource mobilisation and financial allocation. In addition, the advocacy component can build the capacity of leaders to become advocates themselves and speak out on issues pertinent on funding priorities, policy change and addressing social barriers.

Social mobilisation engages and supports participation of institutions, community networks, social/civic and religious groups to raise demand for or sustain progress towards a development objective by strengthening participation in activities at the grassroots level (UNICEF 2008).

The backbone of developing the social mobilisation component of a communication strategy comes from a combination of data, participant and behavioural analyses (FAO 2010b; UNICEF 2008), as well as community input. Behaviour change communication involves face-to-face dialogue with individuals or groups to inform, motivate, problem-solve or plan with the objective of promoting and sustaining change (UNICEF 2008). In agricultural extension and adoption of technologies, sustaining the change implies attaining a critical mass of adopters of a given desirable action (Rogers 2004), in this case ecological sustainability or package of technology that mediates ecological sustainability. According to public engagement conceptual framework (Devine-Wright 2011), effective communication encompasses the understanding of community’s attitudes and believes. The integration of beliefs and attitudes, and the processes used in the assessment of economic, social and environmental dimensions or substantive sustainability, and procedural sustainability is thus relevant (Del Rio & Burguillo 2009).

South Africa and Kenya are classical examples of countries whose agricultural sector is highly vulnerable to changing rainfall patterns. For instance, the area under irrigation as a result of increase in temperature and evaporation in South Africa is projected to increase by 4% – 6% in the interior hydrological zone and 15% – 30% under the hotter and drier hydrological scenarios (DEA 2013). In Kenya, irrigation is recognised as a critical pillar in food security. Of critical importance is that most of the land mass in South Africa and Kenya is located in arid and semi-arid regions. The two countries represent good case studies of potential salinisation risks in climate change action. The possibility of salinisation risks in adaptation to increasing frequency of extreme weather events such as drought in a number of countries, such as Kenya and South Africa, provides an example envisaged by Asghar et al. (2005) where environmental impacts have the potential to increase the severity of disasters and the need for integrative use of analytical lenses that encompass the precautionary principle, disaster risk reduction and risk communication. The above analyses demonstrate the centrality of development for communication strategies in disaster risk reduction.

### Linkages between development for communication, risk communication and sustainable development

The main motivation for future action in disaster preparedness and mitigation is dependent on *a priori* information received. The information disseminated to households, observed information, information density and information content are thus key factors that directly or indirectly increase household action-taking in disaster preparedness and mitigation (Dennis et al. 2011). This increases discussion, perceived effectiveness or efficacy of recommended mitigation or preparedness actions. Though no single generic view of risk communication suffices (Wardman 2008), risk communication is generally defined as the process of disseminating information among interested parties about the nature, magnitude, significance and control of the risk (Covello 1992), dialogue between communication and stakeholders (Palenchar & Heath 2007) and ongoing risk monitoring (Coombs 2012).

Risk communication action raises people’s awareness about given risks, enabling them to be party to individual and collective risk decision-making (Rogers 2004; Stirling 2005). Risk communication plays an integral role in shaping individual risk perceptions as well as risk aversion or reduction behaviours. Risk communication is also a crucial element in risk management processes, as it enables actors to recognise and understand risks, identify their roles and jointly engage in monitoring, reduction, mitigation and recovery efforts (Sato 2015).

As risk communication focuses on the characteristics that impact risk perception and behaviour, it can be appraised through elicitation of the degree of dread associated with a particular risk and the public’s familiarity with the risk (Slovic 2000). Hence, engaging the public in risk appraisal facilitates the correction and adjustment of people’s dispositions and risk behaviour (Stirling 2005). Familiarity and the corresponding dread is used in categorisation of risks into routine, uncertain and controversial risks, respectively (Covello 1992). In this categorisation, the less understood risks are referred to as uncertain risks. Risks that require discussion on values, lifestyles and world views are referred to as controversial risks. Ecological degradation risks could be classified as uncertain risks. In this article, the risk categories are conceptualised on routine-controversial-uncertainty risk continuum.

The risk message model, the risk dialogue model, governability risk model and risk field communication model have been explored in detail by Wardman (2008). The risk message model is about one-way provision of information about the nature of risks (Jaeger & Renn 2001). In the risk message model, it is assumed that people communicate to convey information which may be used by others to enhance their understanding about a given risk. The model focuses on providing or disseminating information about risks which does not necessarily lead to greater understanding (Wardman 2008). Further, it does not consider the effect of complex social networks on expectations, commitment and understanding and co-construction of risk messages and meanings (Jaeger et al. 2001; Horlick-Jones 2008). The mechanistic nature of risk message model (sender–receiver model without engagement or interaction) limits participation thus excluding knowledge from risk message recipients. Such asymmetry reduces the likelihood of the recipient accepting or adopting a particular risk proposition or viewpoints advanced by the experts (O’Neill 2002). In addressing such drawbacks, risk dialogue, risk governability and risk field model have been suggested.

Risk dialogue model seeks to promote participation and inter-subjectivity with an objective of overcoming linearity weaknesses associated with risk message model (Jovchelovitch 2007). Hence, it utilises a two-way communication model to incorporate interests of all actors involved in a particular risk issue. Under the model, the actors are regarded equally and treated like partners. The approach nurtures responsibility and allows for debates as well as collaborative interaction in risk management (Fischhoff 2005). The inter-subjective understanding of risk allows for consensus building between citizens (Jovchelovitch 2007).

The risk field model considers agency in terms of the strategic conduct and interests of competing social actors (Wardman 2008). This is in an attempt to secure legitimacy and enhance trust in risk management and regulatory governance (Pidgeon & Rogers-Hayden 2007). Risk governance model includes interventions that empower individual capacities for self-control, shaping actors and their own sense of self- and self-interest in managing particular risks (Rose 2003). It interrogates how rules that govern the field of possible activity are structured and bear upon the dispositions and behaviour of those concerned. In principle, it focuses on how education and policy discourses can be utilised to mobilise and influence risk dispositions and behaviour of individuals and the transfer of the agency of environmental and health risks control and enforcement from the state to an individual (Rose 2003). As communication for development focuses on use of communication processes, methods and tools to empower people to advance towards full awareness of their situations and their options for change (FAO 2010b), its role is significant in the mitigation of environmental externalities associated with adaptation to climate change in the agricultural sector.

Though risk communication can be used in creating awareness about risks, it has some drawbacks, such as linearity that renders them ineffective (Evans et al. 2018; Servaes & Lie 2013). As the communication for development is concerned with re-engineering diffusion research into a process of innovation, identifying the problem, stakeholder mapping, stakeholder engagement, critical evaluations and reviews as well as addressing the social costs in a participatory manner are some of the prerequisites in re-engineering the process (Leeuwis 2004). We argue that communication for development lenses can be utilised in enhancing the robustness and utility of risk communication in adaptation planning.

The four main elements in the innovation-diffusion model (Rogers 2004) are new ideas or the innovation (in this article adaptation measures and correlated environmental risks), communication channels, time and the social system. Diffusion is facilitated through a communication process by which participants’ access and share information with one another to reach a mutual understanding, form attitudes and implement the idea. Forming attitudes towards an innovation is a mental process in the decision-making and implementation process (Rogers et al. 2007). Disposition about ecological risks, risk behaviour outcomes and agency are thus akin to innovation-diffusion process. Risk communication can thus enhance adoption of positive risk behaviour and technology in the abatement of environmental and health risks (Rogers 2004).

### The proposed conceptual framework

A common feature of contemporary natural resource management issues is the underlying uncertainty in accounting for the problems (such as externalities) and predicting the effect of a particular management strategy. These uncertainties are, in part, a product of the growing emphasis on long-term, multi-scalar and integrative aspects of resource management among researchers, community of practice and policy makers (Biggs et al. 2015). We argue that while multi-scalar and existing integrated models are noble, cognitive failure and biases, lack of approaches for effective participation in environmental risk abatement *vis-à-vis* the agency and the accountability dilemma could exacerbate the externalities and the attendant social costs.

Risk communication is strategic to a meaningful collective action in the governance and management of environmental externalities and securing public interest. However, in adaptation to climate change, policy makers and climate change adaptation actors are more concerned with immediate livelihoods needs (Easterling et al. 2007). As cognitive dissonance about a risk, such as salinisation, may result in such probabilities being downplayed or being ignored (Renn 1998), the use of communication for development and social change approaches that encompass dialogue, agency and mobilisation has been suggested as potential tools in social transformation and resilience building (Evans et al. 2018). In human- environment interactions, a risk-reduction-based planning model that incorporates knowledge, attitudes and behaviour at community level has high potential to cure cognitive failures and contribute to social transformation (Volenzo & Odiyo 2018).

Adaptation-Mitigation-Sustainable Development (AMSD) frameworks are an integral part of wider development goals in transition to sustainability and critical in policy formulation, decision-making, governance and behavioural development (Bizikova, Robinson & Cohen 2007). AMSD includes the creation of local implementation pathways that increase opportunities for social learning processes and capacities for effective adaptation and mitigation (FAO 2010b). As the status of soil salinity as well as its temporal variation can be used as an indicator in the monitoring of degradation risks and as a sustainability indicator (Metternicht & Zinck 2003), salinity levels can serve as an indicator of maladaptation in AMSD frameworks.

**FIGURE 1:**
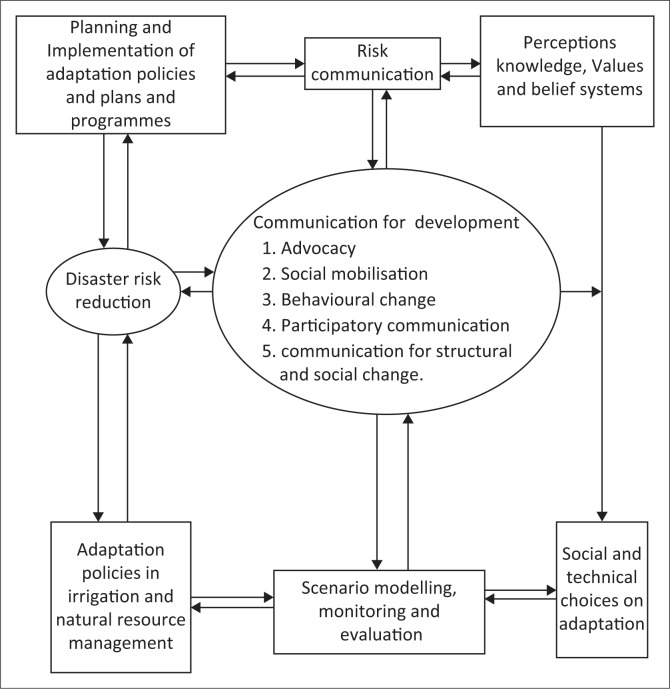
Authors’ conceptual framework on integration between risk communication and sustainable climate change action.

Risk communication focuses on information presentation, persuasion and strategic messages (Heath & O’Hair 2010). Hence, it is critical in closing gaps between what is known by different actors about a particular risk and what needs to be known (Wardman 2008). As knowledge offers insights as to how a system might be managed, farmer’s awareness, perception of environmental risks as well as risk behaviour can be used in modelling environmental impacts in adaptation to climate change. This has the potential in resolving the social-ecological-policy dilemmas. Risk communication can thus be viewed as an integral to risk assessment, risk control, risk monitoring and review planning frameworks for sustainable climate change action. Figure 1 presents the proposed conceptual framework on linkages between risk communication, communication for development and sustainability in climate change action.

## Conclusion

In this article, we make an attempt at exploring the interaction of social mobilisation, advocacy and behaviour development pillars on risk communication concerning the nature, magnitude, significance and control of present and emerging risks in smallholder farmer adaptation to climate change through irrigation. We extended our argument around the concept of maladaptation in smallholder farmer irrigation adaptation practices to suggest an innovative support system for social transformation through communication approaches.

Managing environmental risks in climate change action inadvertently touches on governance in terms of roles, availing of relevant information, policy and legislative frameworks, risk control guidelines as well as coordination mechanism that are responsive to the present and future needs of society. Assessed against urgent need for adaptation action and the equally important dimension of intergenerational equity is thus a socio-ecological dilemma. However, cognitive failure and divergence about the social, economic and environmental dimensions of the sustainability portend a serious challenge in the design of integrative frameworks envisaged under sustainable development agenda.

Though system thinking, nexus and multidisciplinary paradigms provide for coherent planning and sound analytical frameworks, they are inadequate in addressing cognitive failure. This could amplify the adverse impacts of climate change on livelihoods and the environment. Accordingly, innovations in governance, planning and implementation are critical. Strategic and systematic management of underlying natural resource degradation risks in adaptation technologies calls for the integration of communication for development and innovative use of the related concept of risk communication. Implicitly, there is a need for robust social learning analytical frameworks and mechanisms for the integration of knowledge, attitudes and behaviour into climate change policy and implementation discourses. We argue that mainstreaming risk communication approaches into climate change action can broaden the utility of existing analytical frameworks on risk assessment and sustainable development discourses.

Risk communication approaches could be applied in the assessment of farmer awareness, attitudes, knowledge and practices or behaviour (AKAP). Integrating the AKAP findings with results of soil and water analysis across irrigation typologies, agro-ecological and socio-economic environments could, for example, be used in the design of holistic multi-hazard early-warning systems, advisory, capacity building, public awareness, training and education as well as adaptive policy framing, reconstruction and rehabilitation programmes. Risk communication can thus be viewed as an integral to risk assessment, risk control, risk monitoring and review planning frameworks in climate change action and transformation of socio-ecological systems in sustainable development agenda.
